# Static and dynamic mechanical properties and deterioration of bedding sandstone subjected to freeze–thaw cycles: considering bedding structure effect

**DOI:** 10.1038/s41598-020-69270-x

**Published:** 2020-07-30

**Authors:** Sen Chang, Jin-Yu Xu, Er-lei Bai, Guang-Hui Zheng, Xiao-cong Lv

**Affiliations:** 1 Department of Airport Architecture and Engineering, Air Force Engineering University, Xi’an, 710038 China; 2 College of Mechanics and Civil Architecture, Northwest Polytechnic University, Xi’an, 710072 China; 3 Air Support Department of Central Theater, Beijing, 10005 China

**Keywords:** Petrology, Civil engineering

## Abstract

The bedding rock widely exists in nature and its mechanical properties are complex. In this study, the Φ100 mm split Hopkinson pressure bar (SHPB), freeze–thaw(F–T) cycle test system joint with scanning electron microscope and other facilities are applied to investigate the static characteristics, impact characteristics, and damage microstructure of the bedding rock under freezing and thawing conditions. Our experimental results show that under the F–T cycle conditions, the peak point deteriorating path of the static stress–strain curve and the post-peak strain softening curve of the vertical and parallel bedding sandstone specimens have obvious anisotropic characteristics. Parallel bedding specimens have a “pressure bar” effect when loaded. Under the dynamic mechanical test, the peak stress of the vertical bedding specimen is always larger than that of the parallel bedding specimen, and the difference between the two becomes larger while the impact velocity increases. Finally, our microscopic analysis indicates that the main reason for the formation of fissures in the bedding sandstone under the F–T cycle is the cracking of the cement and the shedding of the mineral particles, while the fracture of the mineral particles rarely occurs. The results can provide theoretical guidance for geotechnical engineering in alpine regions.

## Introduction

With the development of human society, people’s multifunctional needs are more abundant and natural resources becomes increasingly scarce. Under these circumstances, geotechnical engineering continues to explore special regions and special environment^1^. Also, the development and utilization of alpine regions is increasingly valued by the geotechnical field. At the same time, more and more problems have emerged during engineering construction and maintenance in the projects involved with rocks in cold area. For example, the rocky water conservancy and hydropower projects in the cold area, transportation engineering, military protection engineering, energy reserve engineering, and civil buildings are all subject to different degrees of F–T damage^2^. Particularly, the bedding rock, of complex mechanical properties, is widely distributed in nature and its understandings are directly related to the safety of many projects^3^.

After several decades of development, a lot of studies are carried out on the damage of freezing and thawing on rock engineering, with fruitful research results. For example, Xu et al.^4^ explored the mechanism of F–T damage of granite using nuclear magnetic resonance technology, and found that when the damage strength exceeds a certain threshold, the accumulation of F–T damage accelerates rapidly. Ozcelik et al.^5^ explored the variation of surface freezing temperature of rock specimens when the number of F–T cycles increased. They found that the freezing temperature of rock surface was related to its ability to resist F–T damage. Furthermore, Sudisman et al.^6^ carried out F–T and tensile tests on three kinds of rocks, and examined the effects of rock types, water content and porosity on the accumulation of F–T damage. In terms of rock mechanics properties, Zhang et al.^7^ simulated the F–T weathering process of red sandstone, and analyzed the variation of rock strength, stress–strain curve and damage propagation mechanical properties with the number of F–T cycles. Kodama et al.^8^ explored the influence of strain rate, water content and temperature on the strength and failure characteristics of rock under freezing from the perspective of dynamic mechanics of rock. Utilizing the improved large diameter (Φ100 mm) SHPB device, Xu et al.^9–13^ investigated the impact mechanical properties of rock under high temperature and confining pressure, achieving fruitful results. As for the microscopic study of rock, Zhou et al.^14^ obtained the transverse relaxation time and nuclear magnetic imaging of granite in F–T environment through nuclear magnetic resonance technology. And based on this, they studied the pore distribution of granite during freezing and thawing. Hori and Morihiro^15^ analyzed the process of freezing and thawing damage of rock and proposed a micromechanical model, which could simulate the damage of rock material in freezing and thawing environment and provide the microscopic analysis data of frozen and thawed rock. Armed with advanced X-ray diffraction, scanning electron microscopy and CT scanning techniques, Ruiz et al.^16^, Park et al.^17^, Kock et al.^18^ tested the pores, fracture structure development and damage accumulation of rock under freezing and thawing conditions, indicating that the microstructural damage of rock has a significant influence on the accumulation of F–T damage. Wang et al.^19–21^ studied the static mechanical properties, dynamic mechanical properties, failure modes, and energy dissipation of red sandstone under freeze–thaw and thermal shock conditions.

Although many previous studies have achieved various understandings on rock performance under freezing and thawing conditions, most of them use isotropic rock as the research object. And few studies involve the development and accumulation of F–T damage of rock with bedding structure. So, in this paper, with the help of Φ100 mm split Hopkinson pressure bar (SHPB) system, electro-hydraulic servo pressure test machine, F–T cycle test system, longitudinal wave velocity detectors, scanning electron microscopes and other equipment, the static and dynamic mechanical properties and microscopic damage of parallel and vertical bedding sandstone under F–T cycles were studied, to provide theoretical support for rock engineering in cold regions.

## Experiments

### Sandstone specimen preparation

The bedding sandstone used in this experiment is wood grain sandstone gathered from a mining engineering in Yunnan, with small and dense mineral particles. The rock is overall pale yellow in the dry state, and the bedding structure is distinguishable by color with the pore strips distributed along the bedding direction. Structurally, the rock belongs to transversely isotropic material. Appraised by the Xi'an Mineral Resources Supervision and Testing Center of the Ministry of Land and Resources, the mineral composition of the rock specimen is composed of 48.6% quartz, 28.4% potassium feldspar, 11.1% illite, 6% dolomite, 2.9% talc, 2.5% calcite and 0.5% diopside. The specimen exhibited a typical crumb structure containing mineral particles, fillers and partial pores.

**Figure 1:**
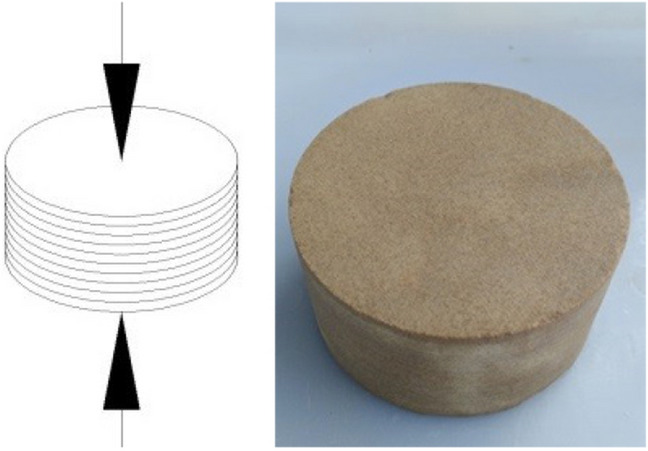
Vertical bedding specimens.

**Figure 2:**
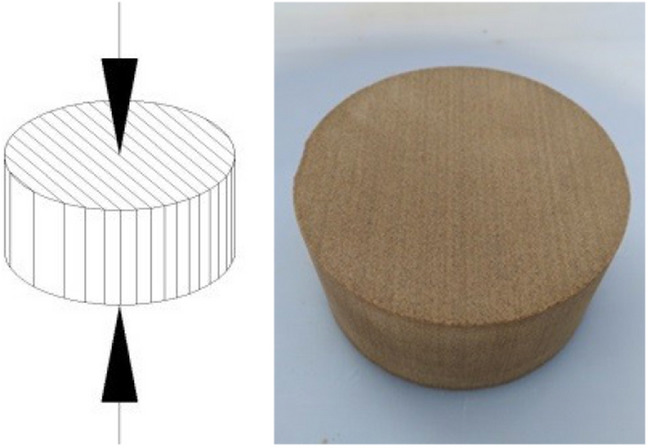
Parallel bedding specimens.

In order to comprehensively and effectively study the development of the mechanical properties, the damage response and failure mode of the bedding rock under F–T cycles, the specimen was processed into two directions ($\alpha { = }0^\circ$ and $\alpha { = 9}0^\circ$) according to the original bedding direction of wood grain sandstone. Wherein, the specimen vertical to the loading direction ($\alpha { = }0^\circ$) was named V-specimens (Fig. 1); The specimen parallel to the loading direction ($\alpha { = 9}0^\circ$) was named P-specimens (Fig. 2).

**Figure 3:**
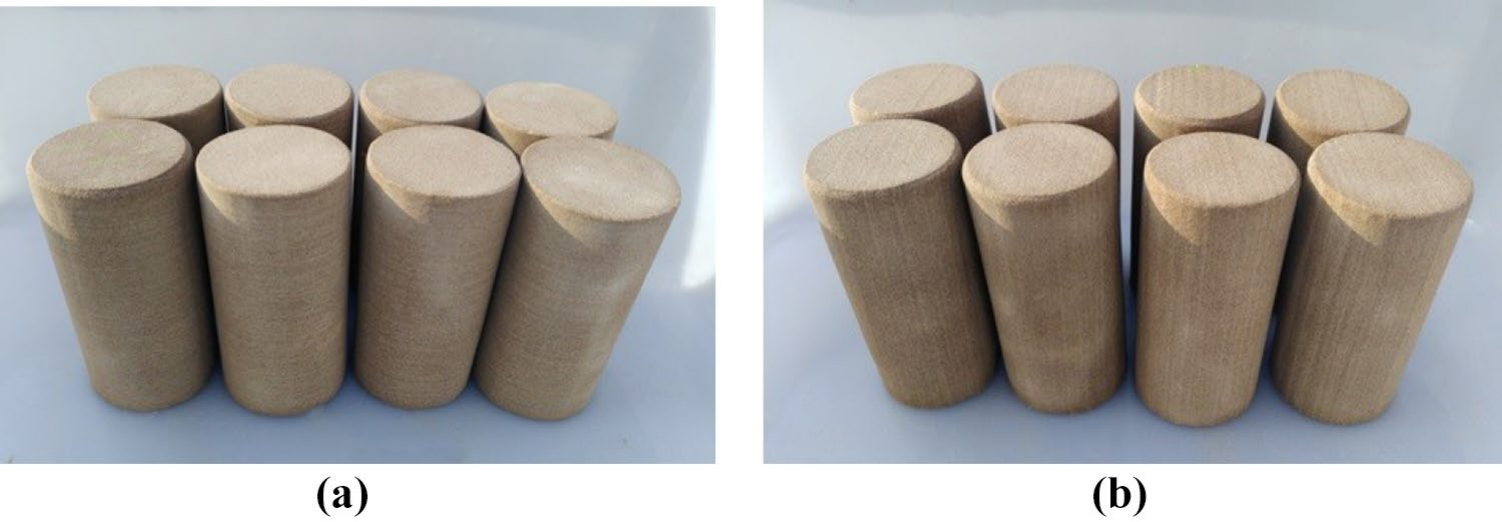
Static compression test specimens: (**a**) vertical bedding; (**b**) parallel bedding.

**Figure 4:**
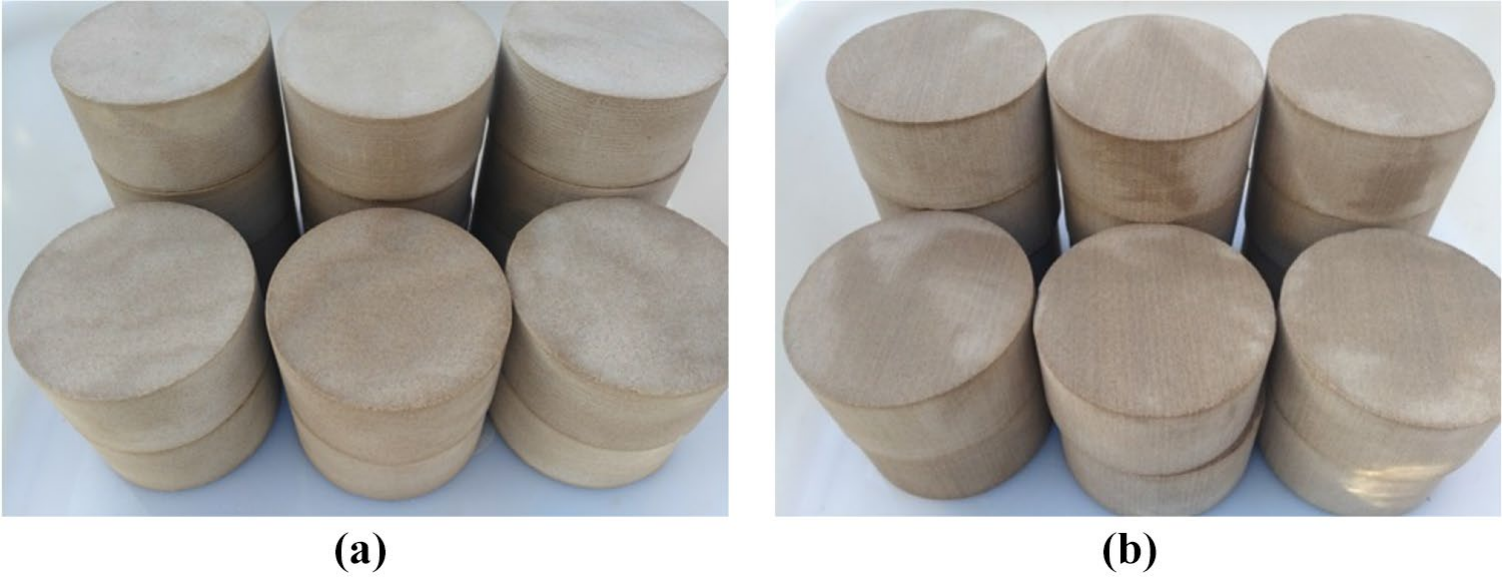
Dynamic compression test specimens: (**a**) vertical bedding; (**b**) parallel bedding.

In addition, according to the previous research results, the wood grain sandstone was processed into two kinds of test specimens with different sizes to study the static and dynamic mechanical properties of the bedding sandstone damaged by F–T cycles systematically:

1. Static compression specimens: According to «GB/T 50266-2013 Engineering Rock Mass Test Method Standard»^22^, the root was divided and polished into a cylindrical standard test piece with a height to diameter ratio of 2:1 (Fig. 3). After processing, the test piece was 100 mm high and 50 mm in diameter; the parallel error of the two end faces was less than 0.05 mm; the vertical error between the end face of the test piece and the axis of the cylinder was less than 0.25°.
2. Dynamic compression specimens: Following the methods and dynamic experimental research results recommended by the International Society of Rock Mechanics (ISRM)^23^, the wood grain sandstone was processed into a standard cylinder of Φ96 mm × 48 mm (Fig. 4). The parallel error of both ends was controlled within 0.5 mm, and the surface flatness was controlled within 0.2 mm.

### F–T treatment of specimen

**Figure 5:**
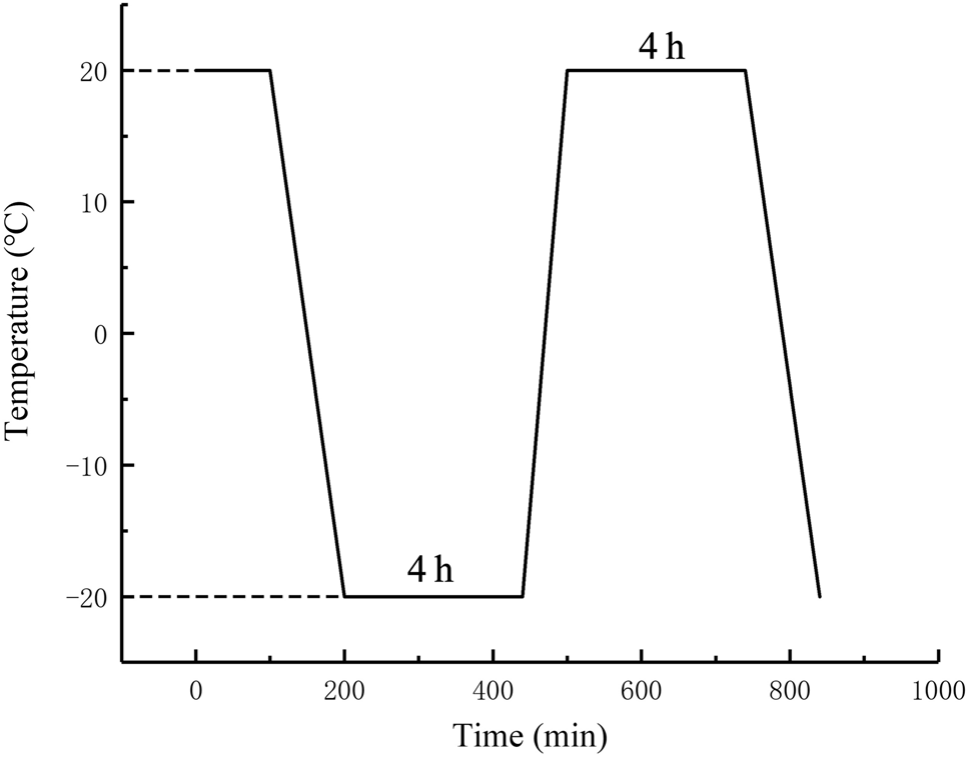
Temperature–time schematic diagrams of F–T cycles.

Firstly, the appearance, volume, quality and ultrasonic signals of the test pieces were screened and measured. The test pieces with large discrete physical indexes were excluded from the following analyses. Subsequently, the screened specimen was placed in a 101-2ASB type electric hot air blower for blast drying at a constant temperature of 107 ± 1 °C for 24 h. Then the specimen was naturally cooled and weighed. After weighing, the drying process was repeated at the same temperature and time duration, and the mass was weighed again after cooling. We repeated the drying–cooling–weighing processes until the mass difference of two measurements was less than 0.1%. Natural cooling was carried out in the drying cabinet. The blast was maintained, and the temperature was turned off to prevent the specimen from absorbing water. Then the dried specimen was put into boiling water to get saturated. The temperature was set to be 100 °C. In the boiling process, the liquid level was ensured to be higher than the specimen constantly. Then, the specimens were kept boiling for 6 h. After the specimen was naturally cooled in the water, it was taken out and put it into the water tank for storage. Finally, F–T test was carried out on the saturated specimen in JCD-40J automatic building material F–T cycle test machine with one F–T cycle procedure set as follows: 4-h freezing in air after the test chamber temperature reaching − 20 °C, followed by a 4-h thawing in water at 20 °C (Fig. 5). During the F–T process, no water existed in the cavity during freezing, and warm water (+ 20 °C) was later injected during melting. The water level was always higher than the specimen, and the temperature change followed almost the same path during each F–T cycle respectively.

And in order to control the systematic uncertainties, 3 mechanical specimens and 2 mesoscopic observation specimens were tested under each experimental condition. For the static pressure test, the number of F–T cycles of the two types of bedding specimens was set to 0, 10, 20, 30, and 40 times.

### Experiment methods

#### Static compression test

As the most conventional mechanical test, the static compression test can provide strength and deformation parameters. These data can provide fundamental reference for the mechanical properties of rock under various loading environments and loading conditions. Therefore, the static compression tests of the two bedding sandstones were carried out in the HYY type electro-hydraulic servo pressure tester at a loading mode of 20 kN/min. The HYY type electro-hydraulic servo pressure test system consists of a pressure table, a hydraulic pump, and a microcomputer control system. The loading limit of the press was 2,000 kN, and the complete stress–strain curve of the specimen was obtained here directly.

**Figure 6:**
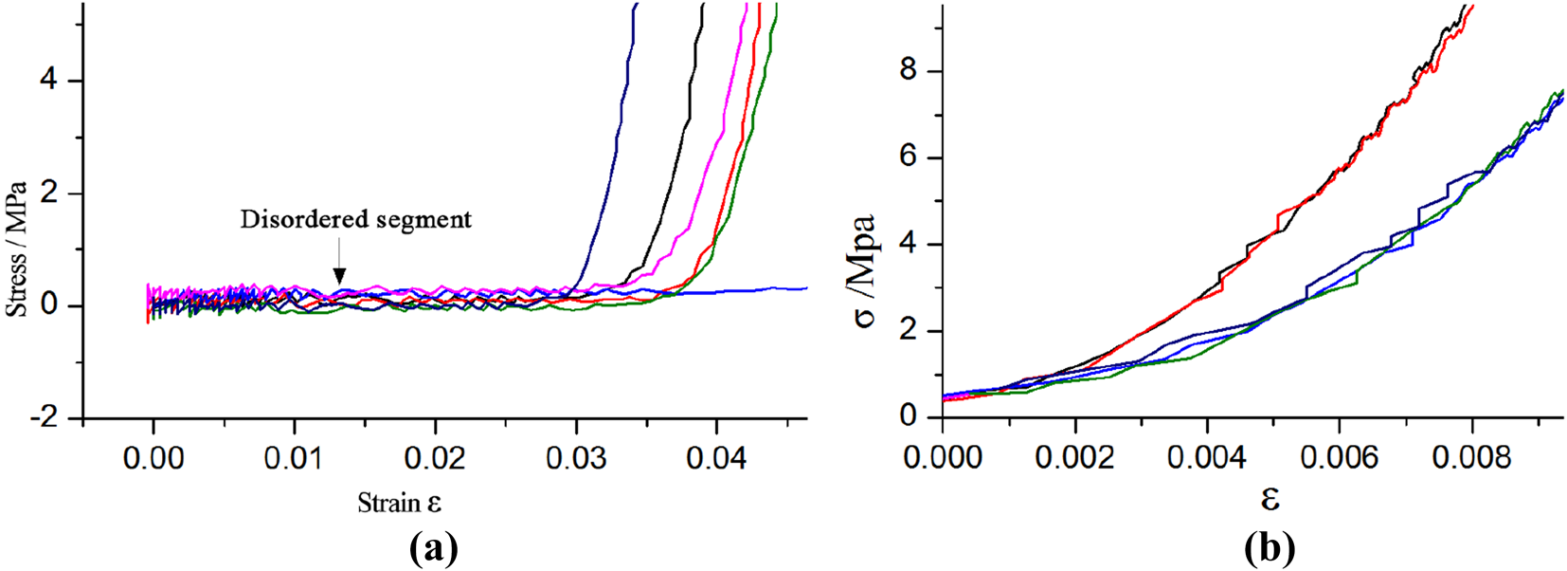
Stress–strain curves of disorder data: (**a**) before treatment; (**b**) after treatment.

When the static compression test was carried out based on the hydraulic servo tester, after placing the sample on the pressure-bearing table, there was a gap between the sample and the upper platen. Therefore, in the initial stage of the static compression test, that is, before the pressure plate and the end face of the sample are closely adhered, the stress–strain curve had obvious fluctuation phenomenon. It couldn’t reflect the true strength deformation property of the material. In order to eliminate the turbulent data generated by the adjustment of the contact between the compressor and the test piece at the beginning of the test, this article referred to the treatment of Nie et al.^24^ and Lu et al.^25^. When the stress–strain curve was processed, the data with the stress greater than 0.5 MPa in the original data was selected as the effective data, and the strain value of the effective data was subtracted from the disorder value to obtain the final stress–strain curve. After processing, there was no large fluctuation phenomenon, which could reflect the strength and deformation performance of the specimen (Fig. 6).

#### Dynamic compression test

To explore the influence of impact velocity, the direction of the bedding structure and the number of F–T cycles on the mechanical properties of the sandstone, the split Hopkinson pressure bar (SHPB) was used for dynamic compression test. The impact test plan is formulated as shown in Table 1.

**Table 1:** Impact test program of the bedding sandstone under F–T cycles.

Layering direction	Freezing and thawing times	Impact velocity/m s^−1^
Vertical layered sample (V)	0, 20, 40	6, 8, 10, 12, 14
Parallel layered sample (P)	0, 20, 40	6, 8, 10, 12, 14

**Figure 7:**
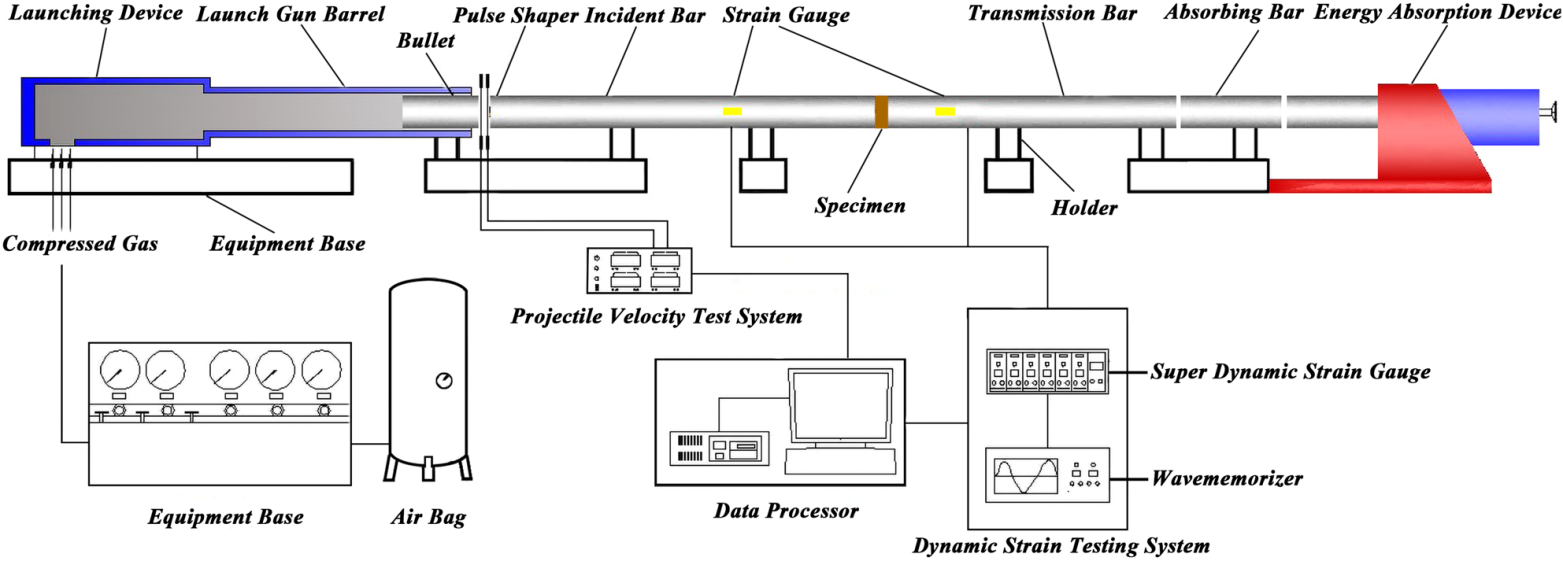
Schematic diagram of Φ100 mm SHPB test system.

The split Hopkinson pressure bar mainly includes an energy storage module, an impact module, and a data acquisition module. The energy storage modules mainly include air compressors, high-pressure tanks (atmospheric packages) and connecting pipes; the impact modules mainly include launching devices, transmission rods, equipment brackets, energy absorption components and consoles (Fig. 7); the data acquisition modules mainly include strain detectors and Bullet velocity measuring instrument. The transmission rod consists of a striking rod (bullet), an incident rod, a transmission rod, and an energy absorbing rod. They are all made of 48CrMoA high-strength alloy steel, with a diameter of 100 mm, an elastic modulus of 210 GPa, a Poisson's ratio of 0.25 to 0.3, and a density of 7.85 g/cm^3^. The strain gauge is attached to the appropriate position of the incident rod and the transmitting rod, which are connected to the data acquisition system. Through dynamic strain tester, the incident, reflection and transmission waveforms in the rod can be collected.

When conducting an impact test, first of all, a high pressure was formed in the high-pressure tank by an air compressor. Then, the console air valve was opened and the preset high-pressure potential energy was instantly converted into the kinetic energy of the striking rod. Then, the striking rod impacted the incident rod at a certain speed, thereby exciting the impact pulse, and then compressing the specimen. The data was collected through a strain gauge attached to the rod and an external strain detector.

**Figure 8:**
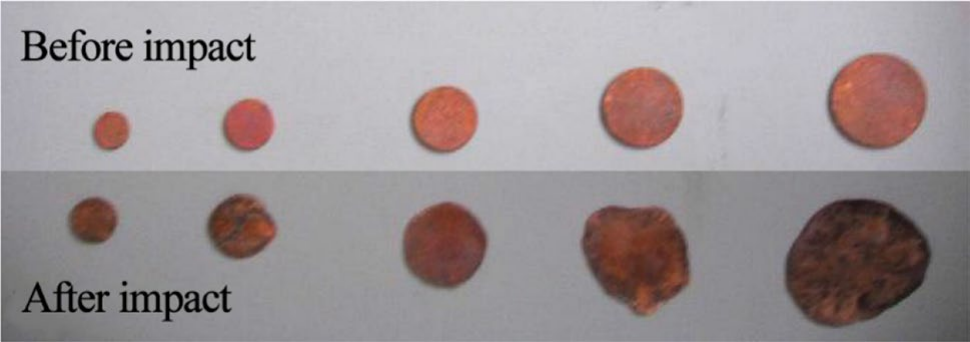
Copper sheet shaper before and after impact.

**Figure 9:**
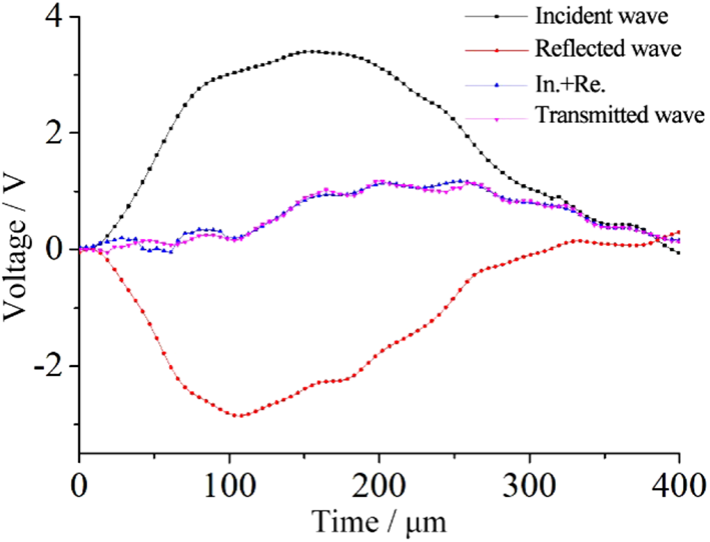
Stress waves in the SHPB impact compression tests.

To ensure dynamic equilibrium and deform uniformity, the annealed copper sheet, 1.0 mm in thick and 10–25 mm in diameter, was used as the wave shaper (Fig. 8). It has shown that the wave shaper can effectively reduce the high-frequency oscillations in the stress pulse. At the same time, it is beneficial to obtain a smooth sinusoid-like incident pulse waveform, extend the rise time of the pulse, and effectively improve the uniformity of the internal stress of the sample. In the impact test, it was confirmed that the sum of the incident wave and reflected wave agreed well with the transmitted wave, reflecting the stress balance of specimens during impact process (Fig. 9).

#### Microscopic test

The EM-30 scanning electron microscope developed by COXEM was carried out to analyze the bedding sandstone specimens under the F–T cycle conditions. Since the rock was a non-metallic material, it was highly insulated from charge. Therefore, in the process of electron beam bombardment scanning of the bedding sandstone specimen, it was easy to form an electron accumulation band on the surface of the specimen, which formed a negative charge region resulting in local discharge phenomenon. This phenomenon would adversely affect the normal scanning of the incident electron beam, indirectly reducing the quality and authenticity of the scanned image. In order to improve the accuracy of electron microscopy, the ETD-800 ion sputter was used to coat gold on the surface of the specimen before electron microscopy forming a conductive loop, avoiding the electron accumulation and negative charge regions.

**Figure 10:**
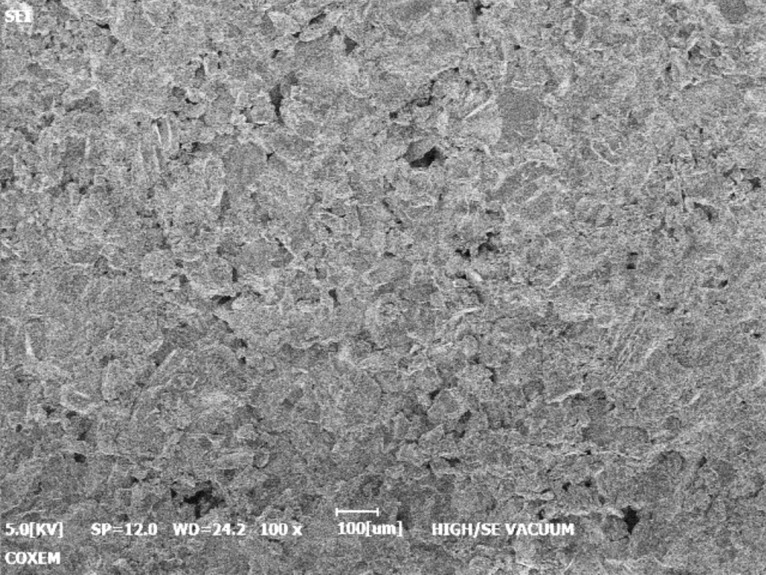
SEM image of wood grain sandstone free from F–T cycles.

Microcracks and surface micro-structure of red-sandstone specimens before accelerated weathering were clear from SEM images as shown in Fig. 10.

## Test results and analyses

### Static stress–strain relationship

**Figure 11:**
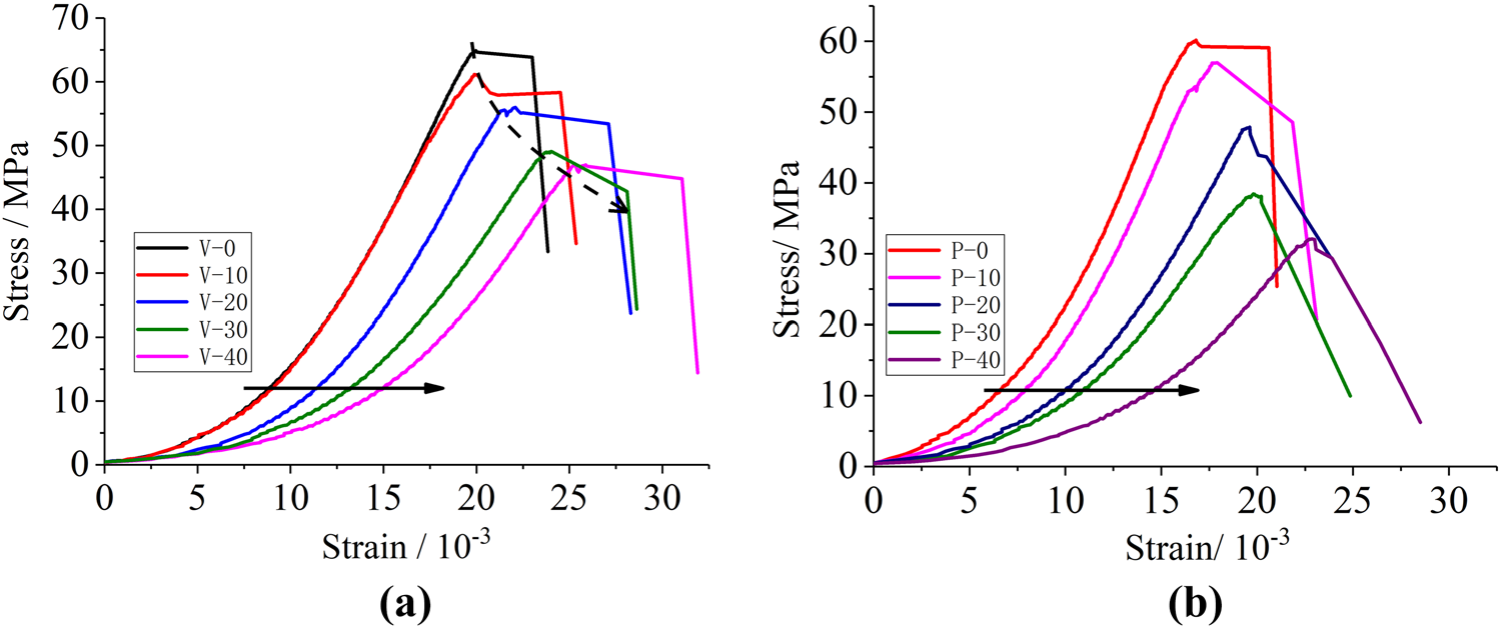
Stress–strain curves under different F–T cycles: (**a**) vertical bedding specimens; (**b**) parallel bedding specimens.

The static stress–strain curve of two bedding sandstones after F–T cycles can be seen in Fig. 11. As the number of F–T cycles increases, the curve stretched to the right and compressed downward, and the range of compressive strain became gradually larger.

Due to the different degree of damage development of the two-bedding specimens during F–T cycles, the structural response of the two-bedding specimens was different when subjected to loads. There were obvious anisotropic characteristics in the morphology of the stress–strain curve of the parallel and vertical specimens after F–T cycle damage. While, the peak point can represent the critical state of the strain. And the strain softening section is a crucial stage in the rock failure. The peak point and the strain softening section were separately analyzed below.

#### Peak point deteriorating path

The peak point of the stress–strain curve is an important feature of the stress–strain relationship. In the stress–strain curve of the F–T specimen, the change of the peak point obviously showed the degradation of the performance of rock material in the F–T environment. Moreover, the peak point of the vertical bedding sample had a gentle descending path, significantly different from parallel bedding specimens. The descending path of the parallel bedding sample was approximately stepped. In other words, the peak strain and peak stress of the vertical bedding specimens decreased smoothly with the increase of the number of F–T cycles. While, the peak stress of the parallel bedding specimens dropped faster than the peak strain.

#### Post-peak strain softening curve morphology

After reaching the peak strength, the rock structure had undergone overall damage. As the material deformation continued to increase, the strength decreased rapidly. This phenomenon was called “strain softening”^26^. From the shape of strain softening curve, the structure–load response relationship after rock instability could be obtained. Figure 11 showed that after the vertical bedding specimen reached the peak strength, the stress was slightly reduced and maintained in a certain strain section. Therefore, the stress balance shown in the figure was actually very short and the strain rate of the material was high. For parallel bedding specimens, there was no stress equilibrium section in the curve after the peak point, and the stress decreased linearly with the increase of strain.

### Static mechanical parameters

**Figure 12:**
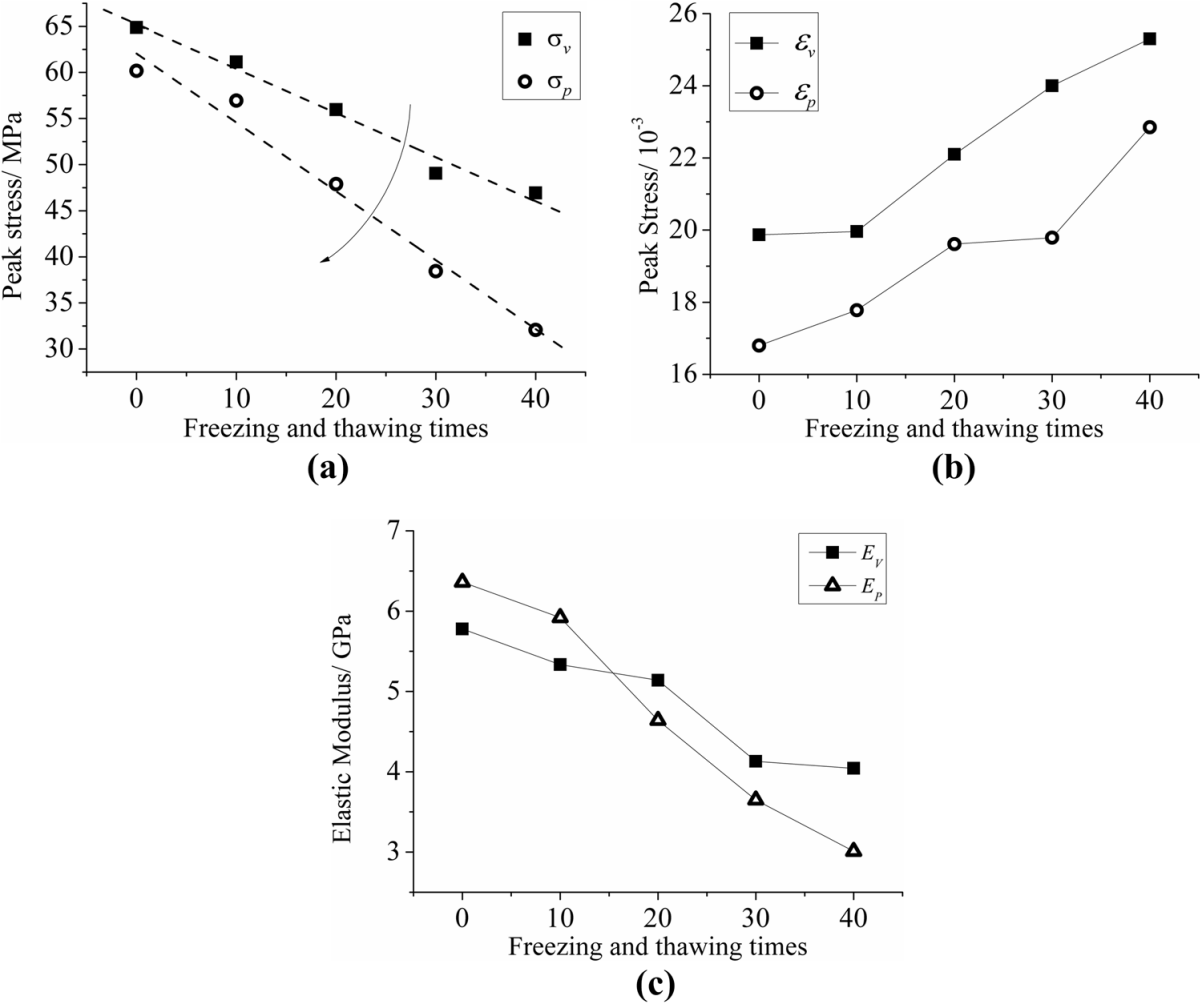
Mechanical parameter curves of two bedding sandstone specimens under different F–T cycles: (**a**) peak stress; (**b**) peak strain; (**c**) elastic modulus.

The mechanical parameters of bedding sandstone under different F–T cycles can be seen in Fig. 12. The analysis showed that as the number of F–T cycles increases, the peak stress and elastic modulus of the bedding sandstone specimen decreased gradually, and the peak strain increased gradually. Moreover, the mechanical parameters of the parallel bedding specimens decreased more rapidly than those of the vertical bedding specimens under the F–T cycle conditions. After 40 F–T cycles, the peak stress of the parallel bedding specimen decreased by 47%, and the elastic modulus decreased by 53%. In comparison, the peak stress of the vertical bedding specimen decreased by 28% and the elastic modulus decreased by 30%. Among them, the peak stress and the number of F–T cycles showed a clear linear relationship, and the fitting effect was evident (Fig. 12a). The fitted curves can be expressed as follows:

1 $$ \sigma_{{\text{V}}} { = }65.19 - 0.48{\text{m }}R^{2} { = }0.97 $$

2 $$ \sigma_{{\text{P}}} { = }62.04 - 0.75{\text{m }}R^{2} { = }0.97 $$

There are two reasons for the increase of peak strain with the increase of the number of F–T cycles. On the one hand, the porosity defects inside the specimen are further developed under the action of freezing and thawing cycles. Once subjected to the crushing load, there will be more strain in the compacting stage. On the other hand, due to the softening effect of water on rock minerals, the deformation ability of the specimen under freezing and thawing conditions is enhanced, resulting in an increase in peak strain at the time of specimen failure.

As observed from the study, the development of the F–T damage of the parallel bedding specimens was higher than that of the vertical bedding specimens. Meanwhile, in general, the higher the damage was to the material, the weaker its ability was to resist deformation. However, as shown in Fig. 12c, the elastic modulus of the parallel bedding specimen was larger than that of the vertical bedding specimen before 10 F–T cycles. It was due to the effectiveness of the bedding structure under load.

1. When the parallel bedding specimens were subjected to load instability, the internal structural mechanical effects of the rock were similar to the “pressing rod instability” of each layer^27^. The deformation resistance of the material was fully exerted, and the elastic modulus was greater.
2. When the number of F–T cycles increased, the degree of F–T damage gradually deepened, so that the cumulative damage effect gradually weakened the "contribution" of the bedding structure to the deformation resistance of the specimen In addition, the parallel bedding specimens had higher damage development; thus, after 20 F–T cycles, the parallel bedding specimens had lower elastic modulus than the vertical bedding specimens.

### F–T cycle effect of impact compression

**Figure 13:**
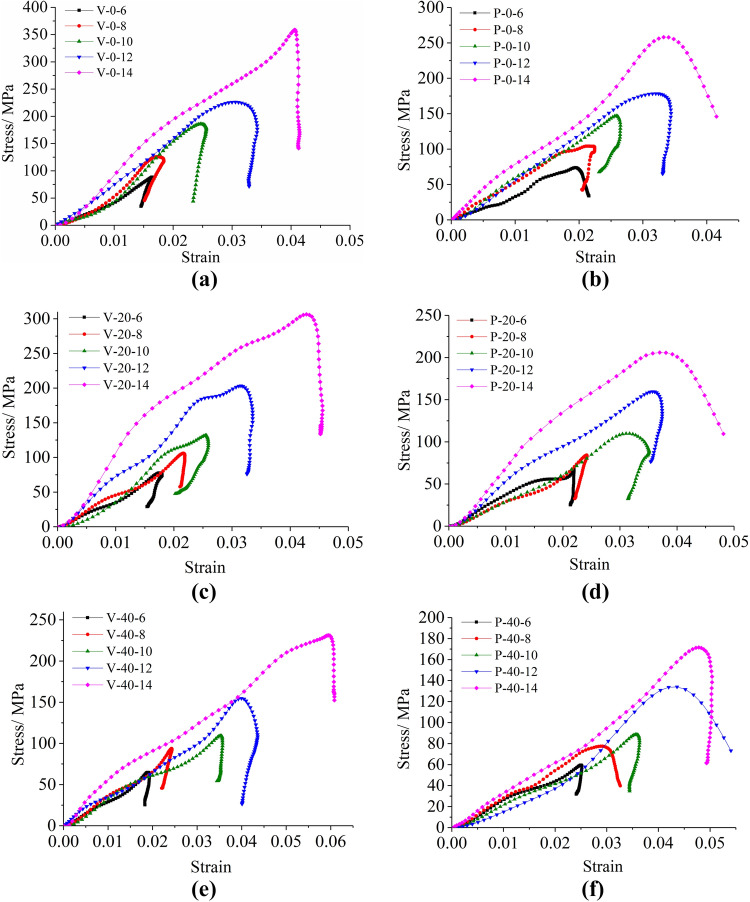
Stress–strain curve of frozen-thawed sandstone specimens under impact: (**a**) Vertical bedding specimens under 0 F–T cycle; (**b**) parallel bedding specimens under 0 F–T cycle; (**c**) vertical bedding specimens under 20 F–T cycle; (**d**) parallel bedding specimens under 20 F–T cycle; (**e**) vertical bedding specimens under 40 F–T cycle; (**f**) parallel bedding specimens under 40 F–T cycle.

Following the “three-wave method”, the collected stress pulse data was processed to obtain the stress–strain relationship of the F–T bedding sandstone under multi-stage impact velocity (Fig. 13). The SHPB test system is a precision test instrument that is susceptible to be disturbed by air pressure, humidity, and external vibration. In the impact test, the violent shock oscillation also has an inevitable effect on the data acquisition performance of the strain gauges. Therefore, it could be seen from Fig. 13 that, unlike the shape of the curve obtained in the static pressure test, there was a certain fluctuation in the stress–strain curve in the impact test.

After comparative analysis of the stress–strain curves of vertical and parallel bedding samples after 0, 20 and 40 freeze–thaw cycles, a common conclusion could be drawn: under the same freeze–thaw cycle conditions, the stress–strain curve showed an overall trend of stretching upward and to the right under the same freeze–thaw cycle conditions, regardless of whether the specimen was vertical or parallel. That is, the peak stress and the peak strain increased as the impact velocity increased.

### Anisotropy analysis

The mineral nature of rock and its structure are the two fundamental factors that control the mechanical properties of rock. The nature of the mineral determines the basic properties of the rock, while the structure of the rock affects the overall performance of the rock. One analog of the relationship between the two is flesh and skeleton. According to the principles of structural mechanics, the presentation of mechanical properties is often closely related to the response relationship between loading force and structure.

For bedding rocks, the bedding structure causes the distribution of mechanical property properties to be uneven. When structures with different properties are regularly distributed in the same rock material, the anisotropic characteristics of strength and deformation performance often occur when the load is applied, which makes the engineering mechanical properties more complicated. The accumulation of damage in the F–T environment increases the complexity of the physical and mechanical properties and directly affects the reliability of the engineering structure. Therefore, it is necessary to analyze this property.

#### Anisotropy of peak stress

**Figure 14:**
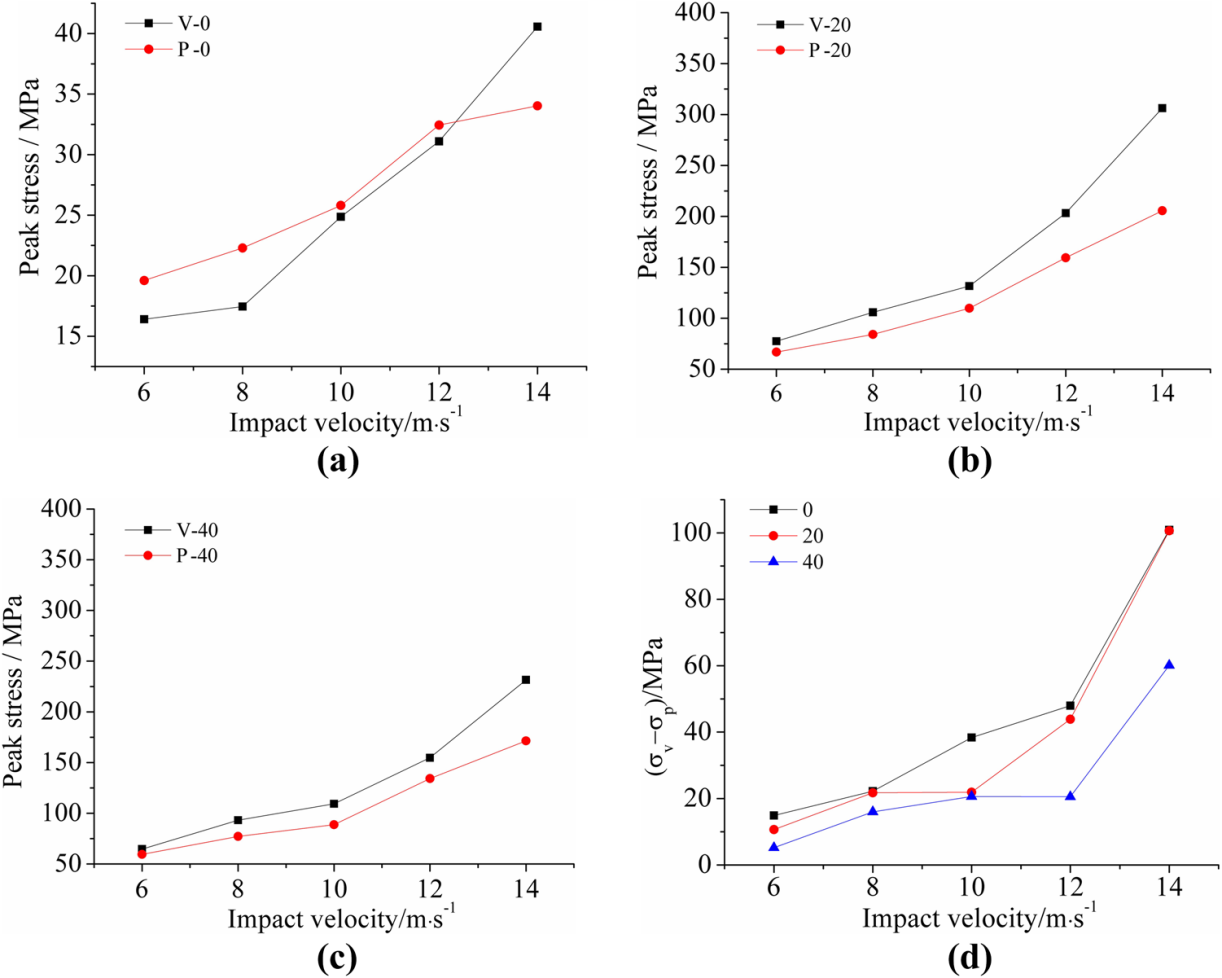
Peak stress of two bedding sandstone specimens under the same number of F–T cycles: (**a**) under 0 F–T cycle; (**b**) under 20 F–T cycles; (**c**) under 40 F–T cycles; (**d**) summary and comparison.

The peak stress curves of the vertical and parallel bedding sandstone specimens at each impact velocity under the same number of F–T cycles are shown in Fig. 14. According to analysis, the following conclusions can be obtained:

1. Figure 14a shows the peak stress of two bedding sandstones without F–T damage. At the same impact velocity, the peak stress of the vertical bedding specimen was always greater than that of the parallel bedding specimen. The results implied that in the absence of differential accumulation of F–T damage, the peak stress of vertical and parallel bedding sandstones was significantly different.
2. Figure 14b,c present the peak stress of the vertical and parallel bedding sandstone specimens after 20 cycles and 40 F–T cycles respectively. The peak stress of the vertical bedding specimens under the same impact velocity was greater than that of the parallel bedding specimens. The result was that in the case of the accumulation of F–T cycles in the specimen, the peak stress of the vertical and parallel bedding sandstones still differed.
3. In comparison of the peak stress of the bedding specimens in two directions from the same number of F–T cycles (Fig. 14d), it could be concluded that as the accumulation of F–T damage increased, the difference in peak stress between vertical and parallel bedding specimens was gradually reduced. The results suggested that under the influence of the accumulation of damage in the F–T cycle, the difference of peak stress caused by the difference in the bedding structure gradually weakened.

#### Anisotropy of peak strain

**Figure 15:**
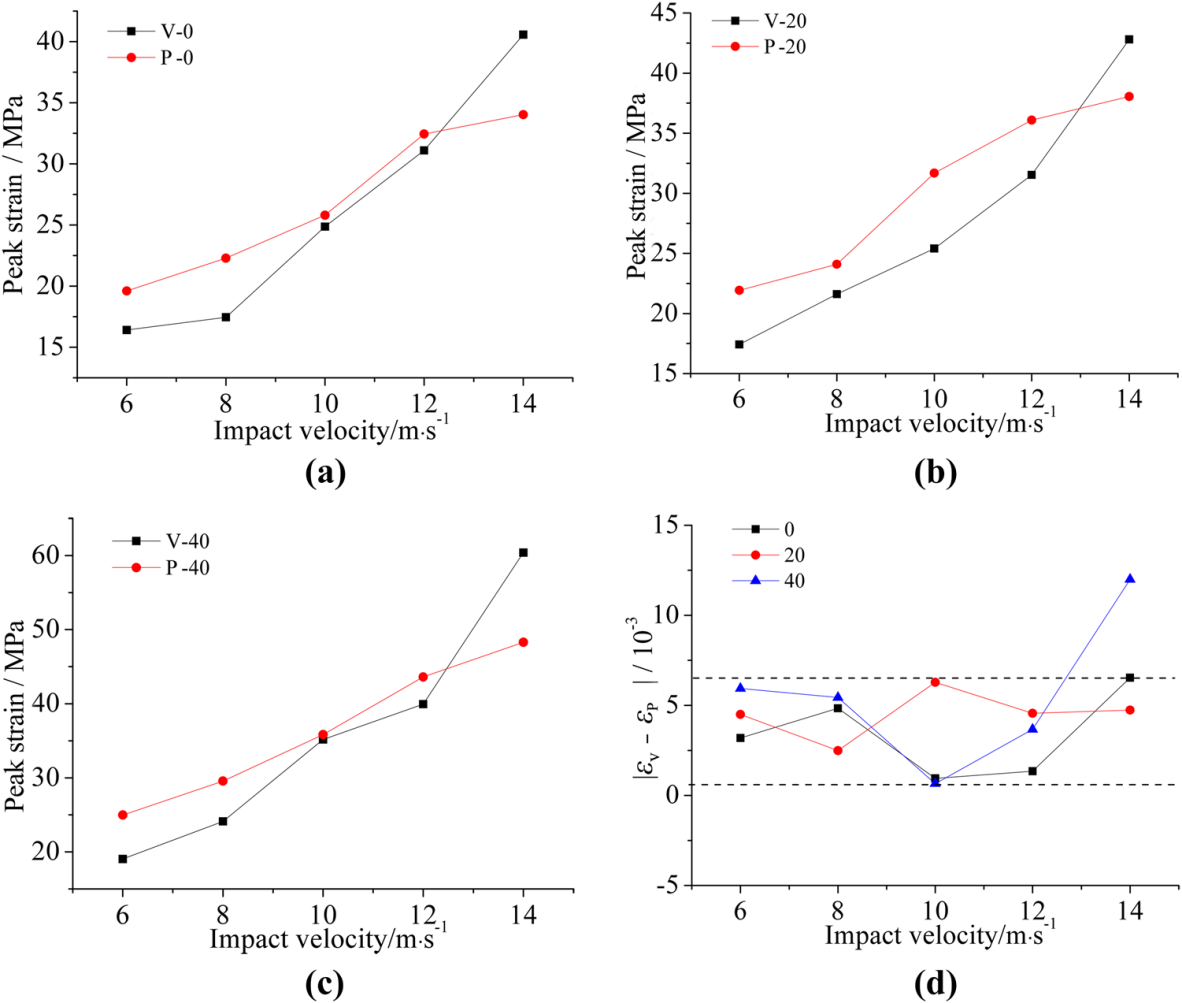
Peak strain of two bedding sandstone specimens under the same number of F–T cycles: (**a**) under 0 F–T cycle; (**b**) under 20 F–T cycles; (**c**) under 40 F–T cycles; (**d**) summary and comparison.

In order to analyze the influence of the bedding structure on the deformation properties of the specimen, the peak strain curves of the bedding sandstone specimens in the two directions under the same F–T cycles were plotted in Fig. 15. After analysis, we come up with the following conclusions:

1. Independent of the number of F–T cycles, when the impact velocity was low (less than 12 m/s), parallel bedding specimens always had greater peak strain than vertical bedding specimens. However, the peak strain of parallel bedding specimens grew slowly with the increase of impact velocity. Moreover, at an impact velocity of 14 m/s, the peak strain of the vertical bedding specimen was larger than that of the parallel bedding specimen.
2. Figure 15d showed that the difference in peak strain of the vertical and parallel bedding sandstone specimens was relatively stable, and the difference was basically stable between 0.65 × 10^−3^ and 6 × 10^−3^. However, at the impact velocity of 14 m/s, the difference of peak strain of the 40 F–T cycles was large (12 × 10^−3^).

## Analysis of meso-morphology after F–T cycles

In the F–T environment, the rock is constantly subjected to the loading and unloading effects brought about by the pore water phase transformation, which leads to the deepening of the F–T cycling damage. The main manifestation is the pores gradually increase and the structure becomes more and more loose. The analysis of the meso-morphology of bedding sandstone under the F–T environment is helpful to deepen the understanding of the damage mechanism of bedding sandstone under the freeze–thaw cycle.

**Figure 16:**
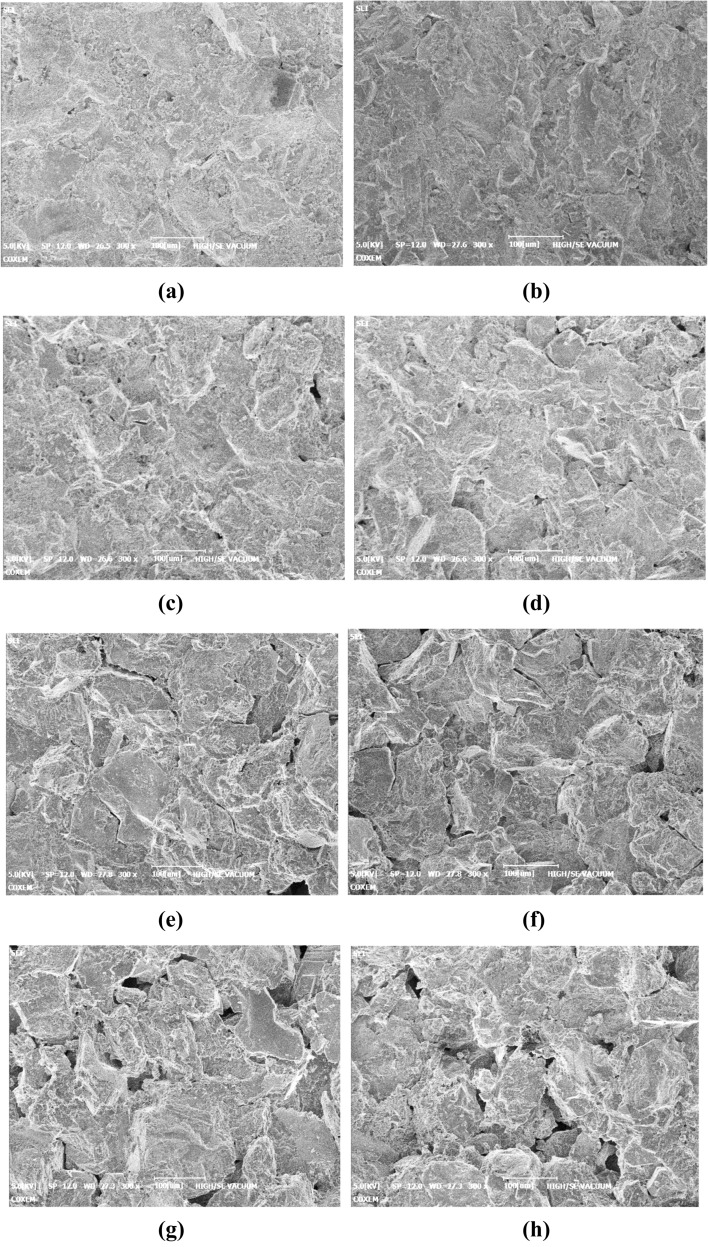

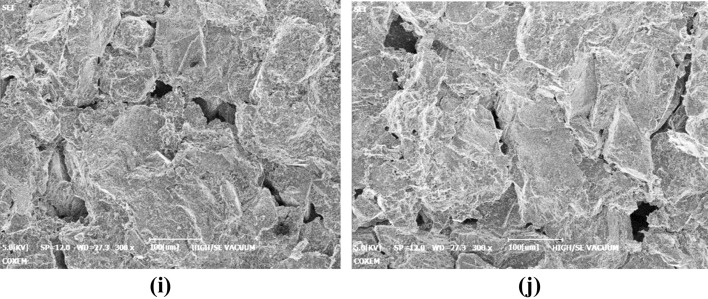
Microscopic morphology of bedding sandstone specimens under different F–T cycles: (**a**) vertical bedding specimens under 0 F–T cycle; (**b**) parallel bedding specimens under 0 F–T cycle; (**c**) vertical bedding specimens under 10 F–T cycle; (**d**) Parallel bedding specimens under 10 F–T cycle; (**e**) vertical bedding specimens under 20 F–T cycle; (**f**) parallel bedding specimens under 20 F–T cycle; (**g**) vertical bedding specimens under 30 F–T cycle; (**h**) parallel bedding specimens under 30 F–T cycle; (**i**) vertical bedding specimens under 40 F–T cycle; (**j**) parallel bedding specimens under 40 F–T cycle.

SEM scanning samples subjected to 0, 10, 20, 30, and 40 F–T cycles were scanned at 300 magnifications to obtain the microscopic morphology of bedding sandstones after different freeze–thaw cycles. The representative micro-morphology of each case can be seen in Fig. 16.

### Mesoscopic differences of bedding rock fragments

At the mesoscale scale, the apparent morphology of the vertical and parallel bedding rock fragments under different F–T cycles was very similar. This similarity was inconsistent with the obvious difference of F–T cycle damage between the two specimens in the static test. This inconsistency existed due to the fact that the accumulation of damage of vertical and parallel bedding specimens during F–T cycle was limited by the relationship between the bedding structure and the size of the specimen. It means that the reason why the two specimens exhibit differential F–T damage accumulation was directly related to size of the specimen. However, the specimens used for SEM electron microscopy were small, about 15 mm × 8 mm × 4 mm, which were expected to have little effect on the development and accumulation of F–T cycles. Therefore, the appearance of vertical and parallel bedding rock fragments was very similar at the mesoscale scale. From this, it could be inferred that the F–T damage accumulation of the bedding specimen had a certain relationship with the correlation between the bedding structure and the specimen size.

### Evolution of bedding sandstone damage under F–T cycles

With the increase of the number of F–T cycles, the mesoscopic appearance of the bedding sandstone has changed significantly. In the case of no freezing and thawing, the cutting marks left during processing could be clearly seen. In other words, the surface morphology was relatively smooth, and the phenomenon of mineral particles being cut off was obvious. Due to the obvious shedding phenomenon of the mineral particles during the F–T cycle, the cutting marks of the surface were not visible when the F–T cycle was completed 10 times. At this time, tiny holes and cracks had appeared on the surface of the rock, but the bond between the mineral particles was still tight. In the subsequent 20, 30, and 40 F–T cycles, the pores further developed and expanded, and the mineral particles were more and more dispersed. Under the 30 F–T cycles, pits and cracks on the surface of the rock surface could be observed due to the falling off of the mineral particles. Moreover, the structural integrity continuously reduced and the pore area and number gradually increased.

### Mechanism of bedding sandstone damage under F–T cycles

According to previous experiments, the crack of the rock under bearing conditions could form by the cracking of the cement between the mineral particles or the direct fracture of the mineral particles. In this study, according to the analysis of the apparent morphology of the bedding sandstone in the F–T environment, the occurrence of the crack under the F–T cycle was mainly caused by the shedding of cement and mineral particles. Our result was closely related to the damage mechanism of the F–T cycle. During the freezing and thawing cycle, the following two aspects cause the cement between the particles to gradually loosen and fall off. The first aspect was the loading and unloading action caused by the repeated phase transformation of the pore water inside the rock. The other aspect was the internal expansion and contraction effect of the rock. While when the number of F–T cycles increases, the liquid water continuously invades defect of the rock and undergoes a water–ice phase change, eventually resulting in significant porosity.

Compared with the vertical bedding sample, the fracture meso-morphology of the parallel bedding sample was smoother, and the mineral particle integrity was higher. The reason is due to different crushing mechanisms of vertical and parallel bedding samples under impact. The bedding structure surface is more vulnerable to failure as a weak link of rock strength. Under the impact, because the layered structure of the parallel layered sample was parallel to the direction of impact compression, the sample was more likely to break along the layered structure surface;

For the vertical bedding sample, the direction of impact compression and the direction of the bedding structure plane were perpendicular to each other. Such a spatial relationship between the load and the bedding structure was conducive to the resistance of the rock to impact deformation. Therefore, the sample needed to consume more energy when it broke. However, when a fracture occurs, a large amount of energy couldn’t be released along the layered structural surface like the parallel layered sample. Meanwhile, the non-layered surface part with a higher bearing capacity of the sample bore a large amount of energy release. Therefore, the microscopic morphology of the specimen under the impact had more debris and fracture of mineral particles.

## Conclusions

The static mechanical properties, dynamic mechanical properties, and damage microstructure of the bedding rock under F–T conditions were tested in this work to study the deterioration of bedding sandstones against F–T cycles. In addition, the meso-morphology and the damage mechanism of bedding sandstone after F–T cycles were studied based on SEM images of fracture surfaces. The following conclusions can be drawn from this study:

1. Under the F–T cycle conditions, the peak-point deterioration path of the stress–strain curve and the post-peak strain softening curve of vertical and parallel bedding sandstone specimens had obvious anisotropy characteristics. With the increase of the number of F–T cycles, the peak stress and elastic modulus of the bedding sandstone specimens gradually decreased, and the peak strain gradually increased. Compared with vertical bedding specimens, the mechanical parameters of parallel bedding specimens deteriorated faster under F–T cycles. The bedding structure had a “pressure bar” effect when the parallel bedding specimen was loaded. Before 10 F–T cycles, the elastic modulus of the parallel bedding specimen was larger than that of the vertical bedding specimen. As the number of F–T cycles increased, the effect of “pressure bar” effect weakened, and the elastic modulus of the vertical bedding specimen was larger than that of the parallel bedding specimen.
2. The peak stress of the bedding sandstone increased with the increase of the impact velocity. The larger the impact velocity was, the faster the peak stress increased; the more F–T cycles were experienced, the smaller the peak stress was. Under the same conditions, the peak stress of the vertical bedding specimen was always larger than that of the parallel bedding specimen, and the difference between the two was more obvious with the increase of the impact velocity. However, when the number of F–T cycles increased, the peak stress difference of the two bedding specimens gradually decreased, and the peak strain increased with the increase of the impact velocity. For vertical bedding specimens, the peak strain under 40 F–T cycles was always significantly greater than that under 0 and 20 F–T cycles; however, the peak strain under 0 and 20 F–T cycles was not significantly different. For parallel bedding specimens, the peak strain increased significantly as the number of F–T cycles increased.
3. Through microscopic analysis, the main reason for the formation of fissures in the bedding sandstone under F–T cycles was the cracking of the cement and the shedding of the mineral particles, and the fracture of the mineral particles rarely occurred. For the parallel bedding sample, the fracture of the parallel bedding sample was smoother and the mineral particle integrity was higher. The sample had a tendency to crack along the surface of the layered structure. For the vertical bedding sample, the spatial relationship between the bedding structure and the load was conducive to help the rock resist impact deformation.
